# Supplementary material for: Construct Validity and Reliability of the Work Environment Assessment Instrument WE-10
Source: Int J Environ Res Public Health. 2020 Oct 9;17(20):7364. doi: 10.3390/ijerph17207364 (PMC7600521; doi:10.3390/ijerph17207364)
Supplement: Supplementary file 1 [file ijerph-17-07364-s001.pdf]

International Journal of
Environmental Research and Public Health

MDPI

Assessment of the Work Environment

Assessment scale of the WE-10 (Work Environment)

Instructions

This questionnaire concerns how you feel regarding the WE-10. It assesses questions on your **quality of life, quality of work life,** and **organizational climate**. Please, answer all of the questions. If you are not sure about your answer to a question, please choose the alternative that seems the most appropriate to you. We are asking how satisfied you are with several aspects of your life and work in the **past two weeks**. Select one of the alternatives and circle the number that best represents your opinion.

1. Health:

1.1. Regarding your health, in general, how do you feel?

Very unsatisfied	Unsatisfied	Neither satisfied nor unsatisfied	Satisfied	Very satisfied
1	2	3	4	5

1.2. Regarding your disposition (energy, liveliness, vitality), how do you feel?

Very unsatisfied	Unsatisfied	Neither satisfied nor unsatisfied	Satisfied	Very satisfied
1	2	3	4	5

1.3. How do you feel about your physical health?

Very unsatisfied	Unsatisfied	Neither satisfied nor unsatisfied	Satisfied	Very satisfied
1	2	3	4	5

1.4. How do you feel about your practice of physical activities?

Very unsatisfied	Unsatisfied	Neither satisfied nor unsatisfied	Satisfied	Very satisfied
1	2	3	4	5

1.5. Regarding your pain and/or discomfort and/or fatigue (tiredness), how do you feel?

Very unsatisfied	Unsatisfied	Neither satisfied nor unsatisfied	Satisfied	Very satisfied
1	2	3	4	5

2. Emotional and Psychological

2.1. How do you feel about your mental/psychological health?

Very unsatisfied	Unsatisfied	Neither satisfied nor unsatisfied	Satisfied	Very satisfied
1	2	3	4	5

2.2. How do you feel about your emotional and psychological state?

Very unsatisfied	Unsatisfied	Neither satisfied nor unsatisfied	Satisfied	Very satisfied
1	2	3	4	5

2.3. Regarding your self-esteem, how do you feel?

Very unsatisfied	Unsatisfied	Neither satisfied nor unsatisfied	Satisfied	Very satisfied
1	2	3	4	5

2.4. How do you feel about your motivation?

Very unsatisfied	Unsatisfied	Neither satisfied nor unsatisfied	Satisfied	Very satisfied
1	2	3	4	5

2.5. Regarding positive feelings, how do you feel?

Very unsatisfied	Unsatisfied	Neither satisfied nor unsatisfied	Satisfied	Very satisfied
1	2	3	4	5

2.6. How do you feel about your social life (social level, conviviality, cultural level)?

Very unsatisfied	Unsatisfied	Neither satisfied nor unsatisfied	Satisfied	Very satisfied
1	2	3	4	5

3. Spiritual

3.1. Regarding your spiritual beliefs, how do you feel?

Very unsatisfied	Unsatisfied	Neither satisfied nor unsatisfied	Satisfied	Very satisfied

1	2	3	4	5

3.2. Regarding your personal values giving meaning to your life, how do you feel?

Very unsatisfied	Unsatisfied	Neither satisfied nor unsatisfied	Satisfied	Very satisfied
1	2	3	4	5

3.3. Regarding your personal beliefs granting you the strength to understand and face hardships in your life, how do you feel?

Very unsatisfied	Unsatisfied	Neither satisfied nor unsatisfied	Satisfied	Very satisfied
1	2	3	4	5

4. Sleep and Rest

4.1. Regarding your sleep and rest, how do you feel?

Very unsatisfied	Unsatisfied	Neither satisfied nor unsatisfied	Satisfied	Very satisfied
1	2	3	4	5

5. Work and Life

5.1. How satisfied are you with the influence of your work on your life routine and family life?

Very unsatisfied	Unsatisfied	Neither satisfied nor unsatisfied	Satisfied	Very satisfied
1	2	3	4	5

5.2. How satisfied are you with the impact of your work on your life and leisure?

Very unsatisfied	Unsatisfied	Neither satisfied nor unsatisfied	Satisfied	Very satisfied
1	2	3	4	5

5.3. How satisfied are you with your work schedule, work routine, and rest?

Very unsatisfied	Unsatisfied	Neither satisfied nor unsatisfied	Satisfied	Very satisfied
1	2	3	4	5

6. Work Conditions

6.1. How satisfied are you with the physical work environment (weather, noise, pollution, contamination, cleanliness)?

Very unsatisfied	Unsatisfied	Neither satisfied nor unsatisfied	Satisfied	Very satisfied
1	2	3	4	5

6.2. Regarding the work conditions (wellness/safety) offered by the company, how do you feel?

Very unsatisfied	Unsatisfied	Neither satisfied nor unsatisfied	Satisfied	Very satisfied
1	2	3	4	5

6.3. How satisfied are you with the safety equipment (individual protection (PPE) and collective protection (CPE)) made available by the company?

Very unsatisfied	Unsatisfied	Neither satisfied nor unsatisfied	Satisfied	Very satisfied
1	2	3	4	5

7. Leadership Management

7.1. Regarding the work pace (speed and manner of execution) conducted by the company, how do you feel?

Very unsatisfied	Unsatisfied	Neither satisfied nor unsatisfied	Satisfied	Very satisfied
1	2	3	4	5

7.2. How do you feel about the clarity of the goals, objectives, and activities developed in the company?

Very unsatisfied	Unsatisfied	Neither satisfied nor unsatisfied	Satisfied	Very satisfied
1	2	3	4	5

7.3. How do you feel about the actions and the support provided by your direct superior (boss/leader) in the company?

Very unsatisfied	Unsatisfied	Neither satisfied nor unsatisfied	Satisfied	Very satisfied
1	2	3	4	5

7.4. How do you feel about the performance of your direct superior (boss/leader) in mediating the existing conflicts in the company?

Very unsatisfied	Unsatisfied	Neither satisfied nor unsatisfied	Satisfied	Very satisfied
1	2	3	4	5

7.5. How satisfied are you with the company's personal appreciation for you?

Very unsatisfied	Unsatisfied	Neither satisfied nor unsatisfied	Satisfied	Very satisfied
1	2	3	4	5

7.6. How do you feel about the opportunity for growth (job promotions and/or career plan) offered by the company?

Very unsatisfied	Unsatisfied	Neither satisfied nor unsatisfied	Satisfied	Very satisfied
1	2	3	4	5

7.7. How do you feel about the autonomy that the company grants you to make decisions?

Very unsatisfied	Unsatisfied	Neither satisfied nor unsatisfied	Satisfied	Very satisfied
1	2	3	4	5

7.8. Regarding teamwork in the company, how do you feel?

Very unsatisfied	Unsatisfied	Neither satisfied nor unsatisfied	Satisfied	Very satisfied
1	2	3	4	5

7.9. How satisfied are you with the feedback about your performance (knowing your performance)?

Very unsatisfied	Unsatisfied	Neither satisfied nor unsatisfied	Satisfied	Very satisfied
1	2	3	4	5

8. Remuneration and Functional Assistance

8.1. How satisfied are you with your remuneration (salary plus benefits, profit sharing)?

Very unsatisfied	Unsatisfied	Neither satisfied nor unsatisfied	Satisfied	Very satisfied
1	2	3	4	5

8.2. How satisfied are you with the assistance benefits (food, transportation, medical assistance, dental assistance) offered by the company?

Very unsatisfied	Unsatisfied	Neither satisfied nor unsatisfied	Satisfied	Very satisfied
1	2	3	4	5

8.3. How do you feel about the assistance provided by the company in a problem that you face?

Very unsatisfied	Unsatisfied	Neither satisfied nor unsatisfied	Satisfied	Very satisfied
1	2	3	4	5

8.4. Regarding educational assistance (studies, courses, training, university) offered by the company, how do you feel?

Very unsatisfied	Unsatisfied	Neither satisfied nor unsatisfied	Satisfied	Very satisfied
1	2	3	4	5

8.5. How satisfied are you with the health assistance (physical or mental) offered by the company?

Very unsatisfied	Unsatisfied	Neither satisfied nor unsatisfied	Satisfied	Very satisfied
1	2	3	4	5

8.6. How satisfied are you with the respect to labor rights demonstrated by the company?

Very unsatisfied	Unsatisfied	Neither satisfied nor unsatisfied	Satisfied	Very satisfied
1	2	3	4	5

8.7. How satisfied are you with the norms and rules of your work?

Very unsatisfied	Unsatisfied	Neither satisfied nor unsatisfied	Satisfied	Very satisfied
1	2	3	4	5

8.8. How satisfied are you with the organizational structure provided for the execution of your job?

Very unsatisfied	Unsatisfied	Neither satisfied nor unsatisfied	Satisfied	Very satisfied
1	2	3	4	5

8.9. How satisfied are you with the company's responsibility for the employees?

Very unsatisfied	Unsatisfied	Neither satisfied nor unsatisfied	Satisfied	Very satisfied
1	2	3	4	5

9. Functional Responsibility

9.1. Regarding the responsibility assigned to you (work responsibility), how do you feel?

Very unsatisfied	Unsatisfied	Neither satisfied nor unsatisfied	Satisfied	Very satisfied
1	2	3	4	5

9.2. Regarding your commitment to the company, how do you feel?

Very unsatisfied	Unsatisfied	Neither satisfied nor unsatisfied	Satisfied	Very satisfied
1	2	3	4	5

9.3. Are you satisfied with your ability to carry out your task/job/activity in the company?

Very unsatisfied	Unsatisfied	Neither satisfied nor unsatisfied	Satisfied	Very satisfied
1	2	3	4	5

9.4. Regarding versatility (the possibility to perform several tasks and jobs), how do you feel?

Very unsatisfied	Unsatisfied	Neither satisfied nor unsatisfied	Satisfied	Very satisfied
1	2	3	4	5

9.5. How do you feel about the communication that takes place in the company?

Very unsatisfied	Unsatisfied	Neither satisfied nor unsatisfied	Satisfied	Very satisfied
1	2	3	4	5

9.6. Regarding the information flow (how the communication occurs), how do you feel?

Very unsatisfied	Unsatisfied	Neither satisfied nor unsatisfied	Satisfied	Very satisfied
1	2	3	4	5

10. Personal Relations and Company Image

10.1. How satisfied are you with your personal relationships (friends, colleagues, bosses) in the company?

Very unsatisfied	Unsatisfied	Neither satisfied nor unsatisfied	Satisfied	Very satisfied
1	2	3	4	5

10.2. Regarding discrimination (social, racial, religious, sexual, among others) at your work, how do you feel?

Very unsatisfied	Unsatisfied	Neither satisfied nor unsatisfied	Satisfied	Very satisfied
1	2	3	4	5

10.3. How satisfied are you with your ability to learn new information?

Very unsatisfied	Unsatisfied	Neither satisfied nor unsatisfied	Satisfied	Very satisfied
1	2	3	4	5

10.4. How satisfied are you with the image of the company to its employees and society?

Very unsatisfied	Unsatisfied	Neither satisfied nor unsatisfied	Satisfied	Very satisfied
1	2	3	4	5

10.5. Regarding your identification with the company (work relation, products, and policies), how do you feel?

Very unsatisfied	Unsatisfied	Neither satisfied nor unsatisfied	Satisfied	Very satisfied
1	2	3	4	5
